# Oxygen care and treatment of retinopathy of prematurity in ocular and neurological prognosis

**DOI:** 10.1038/s41598-021-04221-8

**Published:** 2022-01-10

**Authors:** Hyun Goo Kang, Eun Young Choi, Hyuna Cho, Min Kim, Christopher Seungkyu Lee, Soon Min Lee

**Affiliations:** 1 Department of Ophthalmology, Institute of Vision Research, Yonsei University College of Medicine, Seoul, Republic of Korea; 2 Department of Pediatrics, Yonsei University College of Medicine, Seoul, Republic of Korea

**Keywords:** Retinopathy of prematurity, Risk factors, Paediatric research

## Abstract

This retrospective cohort study aimed to investigate the effects of neonatal oxygen care and retinopathy of prematurity (ROP) treatment on ROP-related ocular and neurological prognoses. We included premature infants treated for ROP at a tertiary referral center between January 2006 and December 2019. Demographic and clinical data were collected from electronic medical records. Odds ratios (ORs) of oxygen care- and ROP treatment-related factors were calculated for ocular and neurological comorbidities 3 years after ROP treatment, after adjusting for potential confounders. ROP requiring treatment was detected in 171 eyes (88 infants). Laser treatment for ROP (OR = 4.73, 95% confidence interval [CI] 1.64–13.63) and duration of invasive ventilation (OR = 1.02, 95% CI 1.00–1.03) were associated with an increase in ocular comorbidities, along with a history of neonatal seizure (OR = 28.29, 95% CI 5.80–137.95) and chorioamnionitis (OR = 32.13, 95% CI 5.47–188.74). No oxygen care- or ROP treatment-related factors showed significant odds for neurological comorbidities. Shorter duration of invasive oxygen supply during neonatal care (less than 49 days) and anti-vascular endothelial growth factor injection as the primary treatment for ROP are less likely to cause ocular comorbidities. No association was identified between ROP treatment modalities and the risk of neurological comorbidities.

## Introduction

Retinopathy of prematurity (ROP), a leading cause of childhood blindness, is a proliferative retinal vascular disease exclusive to premature infants^1^. The primary treatment of ROP is gradually changing from laser photocoagulation ^2^ to anti-vascular endothelial growth factor (VEGF) injection^3^, although neurodevelopmental and systemic outcomes are still under investigation^4^. In recent studies, ROP treatment-related ocular and systemic outcomes of these infants, including neurodevelopmental impairments, were comparable between different ROP treatment modalities^5–8^. However, the effects of changes in perinatal risk and neonatal oxygen care have not been comprehensively investigated in tandem with the change in primary treatment of ROP.

The survival rate among extremely premature infants (less than 28 weeks gestation) is increasing^9^. Concurrently, the rate of multiple births from artificial/assisted fertilization has increased^10^ and the rate of age-dependent pregnancy complications is also increasing^11^. Consequently, the pattern of perinatal risk factors has changed over time^12,13^. Changes in perinatal factors, including neonatal care in prematurity, may affect ophthalmic and systemic outcomes of these infants, not limited by ROP treatment factors.

In neonatal care, the optimal approach to oxygen management remains unclear. Lower oxygen saturation levels increase the risk of mortality, while higher targets are associated with increased rate of oxidative stress^14^. Presently, ventilator-related strategies aim for lower oxygen targets and higher arterial carbon dioxide levels^15,16^. A correlation was found between prematurity, oxygen levels, and ROP risk^17–19^. However, little is known about the impact of changing trends in oxygen therapy on ROP-related prognoses.

To determine which factor related to neonatal oxygen care and ROP treatment, is significant to ROP-related ocular and neurological outcomes, we retrospectively investigated demographic and clinical data in infants treated for ROP over a 14-year period and evaluated possible associations.

## Results

A total of 786 premature infants were screened for ROP, and type 1 ROP requiring treatment was diagnosed in 118 infants, with an overall incidence of 15%. Thirty infants were excluded due to ineligibility (17 for incomplete follow-up and 13 for missing data) and a total of 171 eyes (88 infants) were included in the analyses. The mean follow-up period was 46.6 ± 10.3 months.

Baseline clinical characteristics of ROP infants are provided in Table 1. The average gestational age (GA) was 28.1 ± 2.9 weeks and the mean postmenstrual age (PMA) at treatment was 38.85 ± 7.4 weeks. The average birthweight of premature infants was 1186 ± 475 g. ROP were treated most frequently for stage 3 (59%) following by aggressive posterior ROP (APROP) (26%). Among ROP classification, stage 3 at zone 2 with plus disease was most common (51%). Primary treatment of ROP was laser photocoagulation in 50 eyes (30%) and anti-VEGF injection in 121 eyes (71%). The type of anti-VEGF agent used was ranibizumab in 108 eyes (63%), while bevacizumab in 13 eyes (7.6%). The recurrence rate was significantly higher in anti-VEGF treated eyes (28%) compared to laser-treated eyes (9%, P = 0.003), and there was no significant difference between anti-VEGF agents (12% for bevacizumab vs. 16% ranibizumab, P = 0.13). Additional treatment modalities involved laser (26%), anti-VEGF injection (18%), and scleral buckling (3%). No cases progressed beyond stage 3 ROP after primary treatment, except for one case with stage 4 ROP requiring surgical intervention after laser treatment. In addition, all eyes that required additional laser or anti-VEGF injection for ROP reactivation did not progress to advanced stages and eventually achieved full vascularization of healthy retina.

**Table 1:** Comparison of baseline and clinical characteristics of infants treated for ROP.

	N	Percentage
Infants treated for ROP	88	100
**Gestational age (weeks)**		
< 24	5	5.7
24–27	28	32
27–32	43	49
> 32	12	14
**Birthweight (g)**		
< 1000	39	44
1000–1250	16	18
1251–1500	11	13
1501–1750	7	7.9
> 1750	15	17
Sex, male	39	44
Eyes treated for ROP	171	100
**ROP stage**		
Stage 1	2	1.2
Stage 2	23	14
Stage 3	100	59
Stage 4	1	0.6
APROP	45	26
**ROP classification**		
Zone 1, any stage with plus disease	59	35
Zone 1, stage 3 without plus disease	14	8.2
Zone 2, stage 3 with plus disease	87	51
Zone 2, stage 2 with plus disease	11	6.4
**Primary treatment**		
Laser	50	30
Anti-VEGF injection	121	71
Injection agent, ranibizumab/bevacizumab	108/13	63/7.6

*APROP* aggressive posterior retinopathy of prematurity, *PMA* postmenstrual age, *ROP* retinopathy of prematurity, *VEGF* vascular endothelial growth factor.

Perinatal risk factors detected prior to ROP treatment were described in Table 2. Preterm premature rupture of the membranes was most common (27%) of maternal- and birth-related complications, followed by multiple pregnancy (18%). Among systemic comorbidities, intraventricular hemorrhage was noted most frequent (31%), followed by sepsis (9.1%). Neonatal care-related factors were summarized in Table 2. Invasive ventilation was applied in 82 infants for 42 ± 39.4 days, non-invasive ventilation in 65 infants for 11.1 ± 24.1 days, and oxygen supplementation in 84 infants for 54.1 ± 39.1 days.

**Table 2:** Comparison of perinatal risk factors and neonatal care factors of infants treated for ROP.

	N	Percentage or mean ± SD
**Maternal and birth-related factors**		
Preterm premature rupture of the membranes	24	27
Multiple pregnancy	16	18
Artificial fertilization/assisted pregnancy	8	9.1
Chorioamnionitis	7	8
Gestational diabetes mellitus	4	4.5
Pregnancy-induced hypertension	4	4.5
Placenta previa	2	2.3
Maternal pneumonia	1	1.1
Maternal old age	2	2.3
Placenta abruption	1	1.1
HELLP syndrome	1	1.1
**Systemic comorbidities prior to ROP treatment**		
Intraventricular hemorrhage	27	31
Sepsis (including CNS infection)	8	9.1
Hydrocephalus requiring shunt placement	7	8
Infants requiring cardiopulmonary resuscitation	6	6.8
Neonatal seizure	3	3.4
Congenital heart diseases	2	2.3
**Neonatal care-related factors**		
Duration of hospitalization (days)	88	91.7 ± 58.4
Mean number of admissions	88	1.9 ± 1.1
Duration of incubator care (days)	88	60.5 ± 48.6
Mean number of re-intubations	65	0.8 ± 1.0
Duration of invasive ventilation (days)	82	42 ± 39.4
Duration of non-invasive ventilation (days)	65	11.1 ± 24.1
Duration of O_2_ supplementation (days)	84	54.1 ± 39.1
Blood transfusion	69	78

*HELLP* hemolysis, elevated liver enzymes, low platelet count; *CNS* central nervous system; *ROP* retinopathy of prematurity; *SD* standard deviation.

Table 3 presents ocular and neurological outcomes 3 years after ROP treatment. Strabismus operation rate (15%) was higher than other ocular comorbidities. The incidence of epilepsy or neonatal seizure was assessed in 5.8%, cerebral palsy in 4.1%, and neurodevelopmental delay, evaluated with the Denver II test in 3.5%.

**Table 3:** Comparison of ocular and neurological comorbidities 3 years after treatment for ROP.

	N	Percentage
**Ocular comorbidities**		
Strabismus operation	26	15
Macular dragging	10	5.8
Foveal hypoplasia^a^	10	5.8
Nystagmus	9	5.3
Scleral buckle	2	1.2
Late vitreous hemorrhage^b^	1	0.6
Cataract surgery	1	0.6
**Neurological comorbidities**		
New onset epilepsy or neonatal seizure	10	5.8
Cerebral palsy	7	4.1
Delayed neurodevelopment^c^	6	3.5

*ROP* retinopathy of prematurity.

^a^Poorly developed foveal depression as detected on optical coherence tomography.

^b^Vitreous hemorrhage detected at least 1 year post ROP treatment.

^c^Neurodevelopment was evaluated through the Denver II Developmental Screening Test. Cases of delayed neurodevelopment that occurred unrelated to pre-treatment cerebral complications are presented.

### Risk factors for ocular comorbidities 3 years after ROP treatment

Supplementary Table S1 displays the unadjusted ORs (95% CI) in univariable regression analyses for ocular comorbidities 3 years after ROP treatment. Final multivariable regression model after adjusting confounding variables is presented in Table 4. Laser (vs. injection) treatment for ROP had five-fold-increased odds (odds ratio [OR] = 4.732, 95% CI 1.64–13.63) of ocular comorbidities. Longer duration of invasive ventilation (OR = 1.016, 95% CI 1.00–1.03) increased odds of ocular comorbidities with a history of neonatal seizure (OR = 28.292, 95% CI 5.80–137.95), and presence of chorioamnionitis (OR = 32.133, 95% CI 5.47–188.74). APROP was a possible risk factor for poor eye-related outcomes in the univariable analysis but was not included in the final model of multivariable analysis. Low-risk ROP (zone 2 stage 2 with plus) was not associated with a decreased odds of ocular comorbidities.

**Table 4:** Final multivariable logistic regression model of prognostic factors associated with the risk of ocular comorbidities after treatment for ROP.

	Multivariable	
	OR (95% CI)	*P*-value
Primary laser treatment*	4.732 (1.643–13.629)	**0.004**
Duration of invasive ventilation*	1.016 (1.004–1.028)	**0.009**
Chorioamnionitis*	32.133 (5.471–188.740)	**< 0.001**
Pre-treatment neonatal seizure*	28.292 (5.802–137.951)	**< 0.001**

*CI* confidence interval, *OR* odds ratio, *ROP* retinopathy of prematurity.

*Factors with significant *P*-values in the univariable logistic regression analysis (i.e. *P* < 0.10) were included in the multivariable logistic regression with backward elimination (likelihood ratio). The variance inflation factor of all independent variables included in the final regression model was < 10. A *P*-value in bold text indicates statistical significance (i.e. *P* < 0.05).

**Figure 1:**
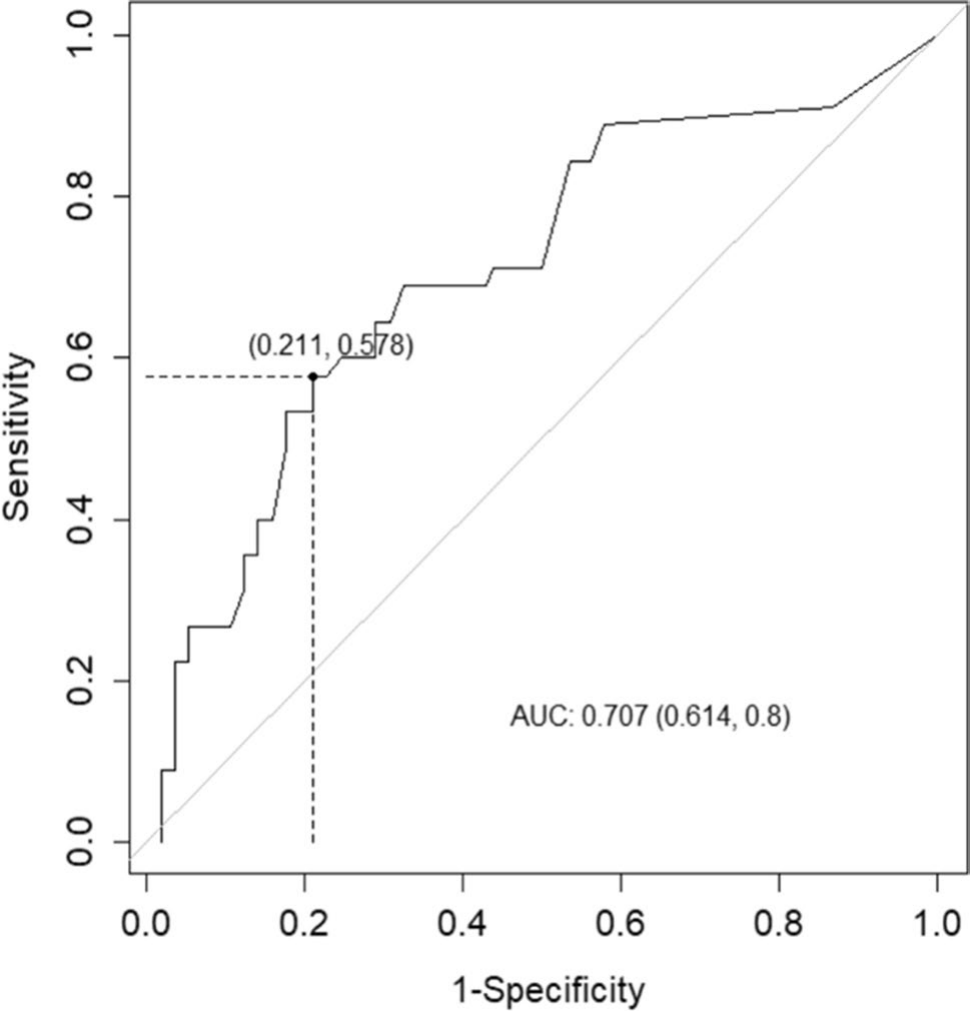
Receiver operating characteristic curve and the Youden’s index to determine the optimal cut-off threshold of invasive ventilation duration that increases the risk of ocular comorbidity.

Analysis of the optimal cut-off for the duration of invasive ventilation, using criterion based on Youden’s index, identified an optical cut-off value of 49 days (Fig. 1). This threshold value in invasive ventilation duration predicts occurrence of ocular comorbidity with 58% sensitivity, 79% specificity, and all with an area-under-the-curve performance of 71%. Additional binary logistic regression modeling of outcomes according to this duration threshold yields statistically significant associations with ocular outcomes (invasive ventilation ≥ 49 days: OR 5.132 for ocular comorbidity, 95% CI 2.44‒10.79; *P* < 0.001).

### Risk factors for neurological comorbidities 3 years after ROP treatment

Supplementary Table S2 displays the unadjusted ORs (95% CI) in univariable regression analyses for neurological comorbidities 3 years after ROP treatment. Final multivariable regression model after adjusting confounding variables is presented in Table 5. The increased odds of treatment modality of ROP (injection vs. laser) for neurological comorbidities was not significant. While bevacizumab had increased odds of neurological comorbidities compared to ranibizumab, this association was excluded in the final regression model. Any factor related with neonatal oxygen care showed no significant odds of neurological comorbidities.

**Table 5:** Final multivariable logistic regression model of prognostic factors associated with neurological comorbidities including neurodevelopmental delay after treatment for ROP.

	Multivariable	
	OR (95% CI)	*P*-value
Birthweight*	0.998 (0.995–1.000)	**0.040**
Pre-treatment intraventricular hemorrhage*	3.836 (1.033–14.242)	**0.045**
Pre-treatment neonatal seizure*	3.836 (1.033–14.242)	**0.045**
Hydrocephalus requiring shunt placement*	16.102 (2.941–88.163)	**0.001**
Incubator care duration*	1.010 (1.001–1.016)	**0.008**

*CI* confidence interval, *OR* odds ratio, *ROP* retinopathy of prematurity.

*Factors with significant *P*-values in the univariable logistic regression analysis (i.e. *P* < 0.10) were included in the multivariable logistic regression with backward elimination (likelihood ratio). The variance inflation factor of all independent variables included in the final regression model was < 10. A *P*-value in bold text indicates statistical significance (i.e. *P* < 0.05).

Higher birthweight (OR = 0.998, 95% CI 0.99–1.00) showed decreased odds of neurological comorbidities. Longer duration of incubator-based care (OR = 1.010, 95% CI 1.00–1.02), a history of intraventricular hemorrhage (OR = 3.836, 95% CI 1.03–14.24), neonatal seizure (OR = 3.836, 95% CI 1.03–14.24), and hydrocephalus requiring shunt placement (OR = 16.102, 95% CI 2.94–88.16) had significantly increased odds of neurological comorbidities. Neither low-risk ROP nor APROP appeared to be associated with neurological comorbidities.

## Discussion

This study shows that less invasive oxygen therapy may result in more favorable ROP-related ocular outcomes in conjunction with primary anti-VEGF therapy for ROP. In addition, a history of chorioamnionitis or neonatal seizure was associated with ocular comorbidities. The choice of ROP treatment (anti-VEGF injection or laser photocoagulation) and anti-VEGF agent (bevacizumab or ranibizumab) appears to have no significant effect on ocular or neurological outcomes, including neurodevelopment.

Among the factors related to neonatal care, the duration of invasive ventilation was associated with an increased risk of long-term ocular comorbidities, whereas ROP severity at the time of treatment did not show a significant association. This result suggests that prolonged invasive ventilation may adversely affect the post-treatment course of ROP. Additionally, the length of incubator care was associated with more common neurological comorbidities. For optimal treatment outcomes in premature infants, our study results indicate that comprehensive multidisciplinary management involving neonatologists, pediatric neurologists, and ophthalmologists is necessary.

Overall, infants who recently received ROP treatment were more frequently associated with artificial/assisted pregnancies. This finding is likely accounted for by increasing maternal age, advances in assisted fertilization techniques, and an increasing frequency of artificial/assisted fertilization. Among maternal- and birth-related factors, the presence of maternal chorioamnionitis was a strong independent risk factor for ROP-associated ocular comorbidities. With improved survival rates of extremely premature neonates, the risk of systemic comorbidities has also increased. Importantly, a history of neonatal seizure was associated with an increase of ocular comorbidities. Therefore, infants with a history of maternal chorioamnionitis or neonatal seizure might require careful screening for ophthalmic comorbidities. As expected, a history of neonatal seizure or brain shunt operation for hydrocephalus was a risk factor for neurological comorbidities, along with a lower birthweight.

Chorioamnionitis has been previously associated with retinal and subretinal vasculopathy. In addition to antenatal and postnatal inflammation, circulatory instability and oxygen satuation fluctuations following inflammation may affect retinal perfusion^20,21^. Prolonged antenatal action of proinflammatory cytokines, major mediator of chorioamnionitis, have been shown to cause delay in retinal vasculature growth, thinning of the choroid, and long-term morphological alterations of the retina^22^. Our findings provide real-world evidence that maternal chorioamionitis is a risk factor for ocular comorbidities. While neonatal seizures are often associated with retinal hemorrhages, they have not been shown to actually cause retinal hemorrhages^23,24^. However, acute symptomatic seizures can occur due to hypoxic-ischemic encephalopathy, acquired structural brain lesions, metabolic disturbances, or systemic infections, which may cause vascular instability and prolonged inflammation that may affect retinal development^25^.

In this study, anti-VEGF injection, compared to laser photocoagulation, was associated with a lower incidence of ocular comorbidities. This difference was most apparent in the number of cases undergoing strabismus surgery that may indicate errors in neurodevelopment as suggested from the pivotal ETROP trials^26^. However, this is likely to be the result of differences in the age and follow-up between the two groups, warranting caution when interpreting the results. The primary method of ROP treatment (whether laser or injection) was not associated with long-term neurologic prognosis, including neurodevelopmental status assessed by the Denver II Developmental Screening Test. The effects of the anti-VEGF agent type (bevacizumab or ranibizumab) were not significant at risk for neurological comorbidity (including delayed neurological development). This is noteworthy because the potential risk for neurodevelopmental delay caused by systemic absorption of intravitreal anti-VEGF is the main cause of concern regarding widespread use in premature infants. When accounting for comprehensive pediatric evaluations and in depth review of medical records, no increased risk stemming from the use of anti-VEGF therapy was shown over laser treatment. Further research is required to confirm the systemic effects of anti-VEGF agents.

In the present study, we noted a significant proportion of infants of GA > 32 weeks treated for ROP in the recent period, which is atypical for infants within this age-group to progress to treatment-requiring ROP. There were no periodic changes in the PMA at treatment, suggesting a faster ROP progression rate among infants of higher GA and indicating that careful observation of neonates may be warranted during the critical PMA window (37–39 weeks).

Recently, interventions appeared to be delivered at an earlier disease stage (ROP classification of zone 2 stage 2 with plus disease), although this difference was not significant. This finding might be due to the simplification of the treatment process from laser photocoagulation to anti-VEGF injection, which might have allowed ophthalmologists greater ease in recommending interventions to eligible neonates with ROP. Furthermore, higher retreatment rates were observed among eyes treated with anti-VEGF. This may be explained by the pharmacokinetics of these anti-VEGF agents and the fact that a higher proportion of eyes were treated with injection for zone 1 disease and APROP^27^.

The limitations of this study include its retrospective nature, lack of a control group, and a homogenous sample, which involved a single ethnic group from a well-developed country with easy access to healthcare. Additionally, we could not account for target levels of oxygen therapy due to the retrospective nature of this study. Instead, the application rate and duration of each oxygen therapy modality were used as variables to consider. Because this study was not a randomized controlled trial, we cannot exclude the possibility of confounding bias caused by heterogeneous and time-varying underlying systemic conditions. Therefore, our presentation of possible associations should be considered with caution, and not be interpreted as a cause-and-effect relationship. Furthermore, although variance inflation factor of all independent variables has been evaluated, collinearity between variables may limit our interpretations.

However, this study was conducted on a relatively large number of treated ROP eyes (N = 171) over a 14-year period at a large tertiary medical center with an average follow-up period greater than 3 years. Considering the paucity of treated ROP cases that considered the neonatal management and the difficulty of regular follow-up with infants, our study results do have clinical importance.

Taking into account the contextual changes in perinatal risk and neonatal care, we have identified some important modifiable factors that may affect the long-term ocular and neurological prognosis of premature infants treated for ROP. In conclusion, shorter application of invasive ventilation during neonatal intensive care unit (NICU) care (less than 49 days) and ROP treatment via anti-VEGF injection have emerged as controllable factors that might improve ophthalmic prognosis among neonates treated for ROP. We were unable to confirm previous concerns regarding a potentially harmful association between anti-VEGF injection or injection drugs and neurological outcomes, in this clinical context.

## Methods

This retrospective study was conducted at Gangnam Severance Hospital, a tertiary referral-based hospital affiliated with Yonsei University College of Medicine. At this hospital, neonates receive care from a multidisciplinary team, including neonatologists, ophthalmologists, anesthesiologists, and other pediatric specialists. Infants requiring ROP treatment were treated at NICUs. This study was based on data extracted from electronic medical records.

Consecutive infants who were treated for type 1 ROP between January 2006 and December 2019 and followed up at least 3 years were included. Neonates were screened for ROP if they met one or more of the following criteria: GA < 32 weeks, birthweight < 1500 g, or unstable clinical course determined by the primary neonatologist based on the applicable protocol at our institution. All ophthalmic examinations were performed by qualified pediatric retinal specialists using the 2005 International Classification of Retinopathy of Prematurity^28^; treatment was indicated for infants diagnosed with type 1 ROP, according to the same criteria as used in a previous study^2^. We further categorized a low-risk ROP type as zone 2 stage 2 ROP with plus disease to determine any possible subtle differences in disease progression and outcomes.

First-line treatment for ROP was chosen after discussion with the infants’ guardians and was performed by pediatric retinal specialists. Although varied due to the specialist’s preference, treatment period, and disease severity, the overall treatment decision could be broadly categorized according to the widening availability of different modalities. Prior to the introduction of anti-VEGF injections for ROP treatment, laser photocoagulation was the treatment of choice. The reporting of reliable safety data regarding anti-VEGF use in neonatal infants has made the injection the primary choice for ROP treatment, especially in less severe cases. In addition, the primary anti-VEGF choice switched from bevacizumab (0.50 mg/0.02 mL, off-label Avastin; Genentech, San Francisco, CA, USA) to ranibizumab (0.20 mg/0.02 mL, off-label Lucentis; Novartis, Basel, Switzerland), after ranibizumab has been shown to have faster systemic clearance and is theoretically safer for use in neonates.

Laser photocoagulation was performed under general anesthesia, using an indirect diode laser system with a 30-diopter (D) hand-held aspheric lens following scleral depression, with power settings adjusted to achieve a pale white burn of the retina. The laser was applied to the avascular area. For intravitreal anti-VEGF injection, either bevacizumab or ranibizumab was injected into the diseased eye under sedation and topical anesthesia. The use of these off-label anti-VEGF agents for treating ROP was approved by the Institutional Review Board. Infants were re-examined the following day and every week thereafter to monitor disease progression. Reappearance of plus disease or progression of ROP stage despite adequate treatment were defined as recurrence. As required, experienced retinal specialists performed additional procedures such as injection, laser, or surgery, including vitrectomy or scleral buckling.

Clinical characteristics of interest included GA, birthweight, ROP-related factors (incidence, severity, treatment age, and modality), and other perinatal factors such as maternal- or birth-related complications, neonatal care (oxygen care type/duration, number of re-intubations, admission duration/re-admission), and systemic comorbidities detected prior to ROP treatment. Invasive ventilation was defined as infants with endotracheal intubation or mechanical ventilation, non-invasive ventilation as infants with face or nasal masks, and oxygen supplementation as infants with oxygen hoods. Target oxygen saturation levels are between 90 and 95% for preterm neonates, while mature infants (GA > 34 weeks) with decreased risk for ROP have a higher target of 97%. The use and duration of each category oxygen were individually tailed in the NICU by trained healthcare professionals; this information is saved to the electronic health records of these infants, and we documented these factors for assessment in this study.

Adverse systemic outcomes were determined through a complete review of patients’ medical records, which included comprehensive examination of follow-up data provided by pediatric specialists; any diagnoses added by the primary pediatrician after ROP treatment were included as systemic comorbidities. Neurological examinations were performed immediately after discharge from the NICU and the Denver II Developmental Screening Test was routinely performed at regular follow-up intervals in the pediatrics department, and those who failed in two or more items of the test were suspected of having neurodevelopmental delay. Ocular comorbidities included any ROP-related ophthalmologic abnormalities (e.g. macular dragging, foveal hypoplasia, optic atrophy, strabismus, late vitreous hemorrhage, cataract, glaucoma, or retinal detachment) that can result in poor visual prognosis detected during ophthalmologic follow-up.

### Main outcomes

The primary outcomes were possible associations between methods of oxygen care and ROP treatment with ROP-related ocular and neurological comorbidities, after adjusting for potential confounders.

### Statistical analysis

Sample size of 84 cases was calculated with type 1 error probability of 0.05 and power of 80%. We performed statistical analyses using the Statistical Package for the Social Sciences software (version 22.0; IBM, Armonk, NY, USA). Listwise deletion was used method in handling missing data. The Kolmogorov–Smirnov test was used to analyze the distribution of the data. Chi-squared tests for categorical variables were used to compare the groups. Logistic regression analysis (backward selection, using the likelihood ratio method) was performed to assess risk factors for ocular and neurological outcomes. Factors with *P* < 0.10 in the univariable logistic regression analysis were included in a multivariable logistic regression model. Optimal cut-off thresholds were analyzed using criterion based on Youden’s index, which maximizes the sum of sensitivity and specificity and also maximizes concordance (RStudio version 1.4.1106; R version 4.0.4; Boston, MA, USA). All data are presented as means (standard deviations) for continuous variables or numbers with percentages for categorical variables. *P* < 0.05 were considered statistically significant.

### Ethics information

Ethical approval was obtained from the Institutional Review Board of Gangnam Severance Hospital (No. 3-2019-0332). Informed consent for treatment was obtained from the patients’ parents or guardians, and the need for consent to be involved in this study was waived due to the retrospective nature of the study. The study adhered to the tenets of the Declaration of Helsinki.

## Supplementary Information

Supplementary Tables.

## Data Availability

Anonymized participant data are available upon reasonable request from Dr EY. Choi. However, data reuse is not permitted.
