# Supplementary material for: Oxygen care and treatment of retinopathy of prematurity in ocular and neurological prognosis
Source: Sci Rep. 2022 Jan 10;12:341. doi: 10.1038/s41598-021-04221-8 (PMC8748614; doi:10.1038/s41598-021-04221-8)
Supplement: Supplementary file 1 — Supplementary Tables. [file 41598_2021_4221_MOESM1_ESM.docx]

Supplemental Material

**Oxygen care and treatment of retinopathy of prematurity in ocular and neurological prognosis**

Hyun Goo Kang^1^, Eun Young Choi^1*^, Hyuna Cho^1^, Min Kim^1^, Christopher Seungkyu Lee^1^, Soon Min Lee^2^

^1^Department of Ophthalmology, Institute of Vision Research, Yonsei University College of Medicine, Seoul, Republic of Korea

^2^Department of Pediatrics, Yonsei University College of Medicine, Seoul, Republic of Korea

***Corresponding author:**

Eun Young Choi, MD

Department of Ophthalmology, Institute of Vision Research, Severance Eye Hospital, Yonsei University College of Medicine

50-1 Yonseiro, Seodaemun-gu, Seoul 03722, Republic of Korea

Tel: +82-2-2228-3586, Fax: +82-2-3463-1049

Email: eychoi@yuhs.ac

**Supplementary Table S1.** An univariable analysis of prognostic factors associated with the risk of ocular comorbidities after treatment for ROP

|  | **Univariable** | |
| --- | --- | --- |
|  | **OR (95% CI)** | ***P*-value** |
| **Gestational age*** | 0.805 (0.706-0.918) | **0.001** |
| **Birthweight*** | 0.998 (0.997-0.999) | **<0.001** |
| **Gestational diabetes mellitus** | 1.091 (0.204-5.829) | 0.92 |
| **Pregnancy-induced hypertension*** | 4.959 (1.135-21.665) | **0.03** |
| **Chorioamnionitis*** | 12.781 (3.378-48.363) | **<0.001** |
| **Pre-treatment intraventricular hemorrhage*** | 4.124 (2.014-8.443) | **<0.001** |
| **Pre-treatment neonatal seizure*** | 9.455 (3.144-28.432) | **<0.001** |
| **Sepsis (including CNS infections)*** | 3.079 (1.082-8.764) | **0.04** |
| **Low-risk ROP (zone 2 stage 2 with plus)** | 0.605 (0.447-2.762) | 0.47 |
| **Aggressive posterior ROP*** | 0.331 (0.129-0.845) | **0.021** |
| **Primary laser treatment*** | 2.134 (1.045-4.356) | **0.04** |
| **Bevacizumab (vs. ranibizumab)** | 0.409 (0.122-1.375) | 0.15 |
| **Retreatment for ROP reactivation*** | 2.167 (0.989-4.744) | 0.05 |
| **Duration of invasive ventilation*** | 1.014 (1.005-1.022) | **0.002** |
| **Duration of non-invasive ventilation** | 1.013 (0.994-1.033) | 0.19 |
| **Duration of O_2_ supplement*** | 1.020 (1.011-1.029) | **<0.001** |
| **Incubator care duration*** | 1.011 (1.003-1.018) | **0.004** |
| **No. re-intubations*** | 2.034 (1.413-2.927) | **<0.001** |
| **Blood transfusions** | 1.482 (0.592-3.709) | 0.40 |
| CI = confidence interval; CNS = central nervous system; OR = odds ratio; ROP **=** retinopathy of prematurity  ^*^Factors with significant *P*-values in the univariable logistic regression analysis (i.e. *P*<0.10) were included in the multivariable logistic regression with backward elimination (likelihood ratio).  A *P*-value in bold text indicates statistical significance (i.e. *P*<0.05) | | |

**Supplementary Table S2.** An univariable analysis of prognostic factors associated with neurological comorbidities including neurodevelopmental delay after treatment for ROP

|  | **Univariable** | |
| --- | --- | --- |
|  | **OR (95% CI)** | ***P*-value** |
| **Gestational age^*^** | 0.729 (0.606-0.876) | **0.001** |
| **Birthweight^*^** | 0.997 (0.995-0.999) | **0.001** |
| **Gestational diabetes mellitus** | 2.224 (0.405-12.104) | 0.36 |
| **Pregnancy-induced hypertension** | 1.840 (0.351-9.639) | 0.47 |
| **Preterm premature rupture of the membranes** | 0.942 (0.370-2.402) | 0.90 |
| **Chorioamnionitis** | 0.880 (0.186-4.174) | 0.87 |
| **History of cardiopulmonary resuscitation** | 1.136 (0.474-2.727) | 0.78 |
| **Pre-treatment intraventricular hemorrhage^*^** | 3.143 (1.064-9.280) | **0.04** |
| **Pre-treatment neonatal seizure^*^** | 3.143 (1.064-9.280) | **0.04** |
| **Hydrocephalus requiring shunt placement^*^** | 4.857 (1.532-15.400) | **0.007** |
| **Sepsis (including CNS infections)** | 1.913 (0.568-6.449) | 0.30 |
| **Low-risk ROP (zone 2 stage 2 with plus)** | 2.446 (0.591-10.125) | 0.22 |
| **Aggressive posterior ROP^*^** | 0.769 (0.289-2.047) | 0.60 |
| **Primary laser treatment^*^** | 2.232 (0.959-5.194) | 0.06 |
| **Bevacizumab (vs. ranibizumab)^*^** | 7.833 (2.298-26.706) | **0.001** |
| **Retreatment for ROP reactivation** | 1.450 (0.558-3.766) | 0.45 |
| **Duration of invasive ventilation** | 1.005 (0.997-1.014) | 0.20 |
| **Duration of non-invasive ventilation** | 1.020 (0.997-1.044) | 0.093 |
| **Duration of O_2_ supplement^*^** | 1.010 (1.000-1.020) | **0.04** |
| **Incubator care duration^*^** | 1.011 (1.003-1.019) | **0.005** |
| **No. re-intubations^*^** | 1.690 (1.172-2.435) | **0.005** |
| **Blood transfusions** | 3.136 (0.700-14.060) | 0.14 |
| CI = confidence interval; CNS = central nervous system; OR = odds ratio; ROP **=** retinopathy of prematurity  ^*^Factors with significant *P*-values in the univariable logistic regression analysis (i.e. *P*<0.10) were included in the multivariable logistic regression with backward elimination (likelihood ratio).  A *P*-value in bold text indicates statistical significance (i.e. *P*<0.05) | | |
